# Nonstructural protein P7-2 encoded by *Rice black*-*streaked dwarf virus* interacts with SKP1, a core subunit of SCF ubiquitin ligase

**DOI:** 10.1186/1743-422X-10-325

**Published:** 2013-11-01

**Authors:** Qian Wang, Tao Tao, Yanhong Han, Xiangru Chen, Zaifeng Fan, Dawei Li, Jialin Yu, Chenggui Han

**Affiliations:** 1 Key Laboratory for Tobacco Gene Resources, Tobacco Research Institute, Chinese Academy of Agricultural Sciences, Qingdao 266101, P. R. China; 2 State Key Laboratory for Agro-biotechnology and Ministry of Agriculture Key Laboratory for Plant Pathology, China Agricultural University, Beijing 100193, P. R. China

**Keywords:** *Rice black*-*streaked dwarf virus*, P7-2, SKP1, Interaction, SCF ubiquitin ligase

## Abstract

**Background:**

*Rice black*-*streaked dwarf virus* (RBSDV), a member of the genus *Fijivirus* within the family *Reoviridae*, causes severe damage to cereal crops in South East Asia. The protein P7-2, encoded by the second open reading frame of segment S7, is conserved among most plant-infecting fijiviruses, but its function is still obscure.

**Results:**

In this study, P7-2 was used as bait in two-hybrid screens of a cDNA library expressing *Zea mays* proteins. It was found that there is a strong interaction between P7-2 and *Z*. *mays* SKP1 (SKP1^Maize^), a core subunit of the multicomponent SCF (SKP1/Cullin1/F-box/Rbx1) E3 ubiquitin ligase. The interaction was then confirmed in leaf epidermal cells of *Nicotiana benthamiana* by bimolecular fluorescence complementation assay. Further investigations indicated that P7-2 also interacts with SKP1 proteins from other plants, including *Arabidopsis thaliana*, *N*. *benthamiana*,*Oryza sativa* and *Saccharum sinense*. The C-terminal fragment of SKP1^Maize^ (residues 97–176) and the middle fragment of P7-2 (residues 79–214) are necessary to sustain the interaction, while the C-terminal putative α-helix domain spanning residues 214–295 of P7-2 greatly facilitates the interaction. Agrobacterium-mediated transient suppression assay showed that P7-2 has no obvious activity to suppress local RNA silencing.

**Conclusions:**

Taken together, our results indicated that RBSDV P7-2 can interact with SKP1 proteins from different plants. This is the first report linking a *Fijivirus* protein to a component of the ubiquitin proteasome system. P7-2 might be a potential F-box protein encoded by RBSDV and involved in the plant-virus interaction through ubiquitination pathway.

## Background

*Rice black*-*streaked dwarf virus* (RBSDV) is an insect- and plant-infecting agent that belongs to the genus *Fijivirus* in the family *Reoviridae*. The virus is transmitted propagatively via the small brown planthopper *Laodelphax striatellus* and causes rice black-streaked dwarf and maize rough dwarf diseases in southeast Asian countries [1-4]. Infected plants show typical stunting, darkening of leaves and waxy white tumors or black-streaked galls along the veins on the underside of leaf blades, sheaths and columns [1,5].

The mature RBSDV virion is an icosahedral, double-layered particle with a diameter of 75–80 nm and consists of 10 genomic double-stranded RNA (dsRNA) segments (S1-S10) [6,7]. Most genomic segments contain one open reading frame (ORF), except S5, S7 and S9, which are bicistronic. Six structural proteins (P1, P2, P3, P4, P8 and P10) and six nonstructural proteins (P5, P6, P7-1, P7-2, P9-1 and P9-2) are encoded by RBSDV genome. P1, P2, P3 and P4 are assigned to be the putative RNA-dependent RNA polymerase, a core protein, a putative capping enzyme and the outer-shell B-spike protein of the virion, respectively, based on their deduced amino acid sequences and molecular masses [6-8]. P8 is the minor core capsid protein and possesses potential active transcriptional repression activity [9]. P10 is the main component of the outer shell of the viral particle and can self-interact to form trimers in solution [5,10]. Both S7 and S9 have two non-overlapping ORFs and encode two nonstructural proteins. P7-1 is involved in the formation of tubular structures, and the secretory pathway and actomyosin motility system in plant hosts are required for its plasmodesmatal localization [5,11]. P9-1, a thermostable, α-helical protein, is a major viroplasm matrix protein and plays a key role for the formation of viroplasms [5,12]. Crystallographic analysis indicated that P9-1 is able to form cylindrical octamers via lateral hydrophobic interactions [13]. Immunoelectron microscopy showed that P5 and P6 are also constituents of the viroplasms. P6 has strong ability to self-interact or bind to P9-1, and P5 is recruited to viroplasms by binding to P6 [14,15]. The functions of the remaining proteins are still largely unknown.

RBSDV P7-2 is a nonstructural protein encoded by the second ORF of segment S7, containing 309 amino acids with a molecular mass of 36 kDa. Interestingly, the gene is conserved among most plant-infecting fijiviruses except *Nilaparvata lugens reovirus* (NLRV), a non-phytopathogenic hopper-borne fijivirus. The counterparts of P7-2 were found in the genomes of all phytopathogenic fijiviruses reported including *Fiji disease virus* (FDV), *Oat sterile dwarf virus* (OSDV), *Mal de Río Cuarto virus* (MRCV), *Maize rough dwarf virus* (MRDV) and *Southern rice black*-*streaked dwarf virus* (SRBSDV). NLRV shares many properties with RBSDV, including the particle structure, terminal nucleotide sequences of genome segments, deduced amino acid sequences, manner of transmission through rice plants and multiplication ability in *L*. *striatellus*[16]. The most distinct biological difference between NLRV and RBSDV is that NLRV is a nonphytopathogenic reovirus, unable to reproduce in rice plants, and it uses rice plants as the transmittable vector [16-18]. Taken together, RBSDV P7-2 and its counterparts were considered to be involved in virus multiplication in plants or viral pathogenicity on plants. Unfortunately, antisera to P7-2 failed to detect the protein in either RBSDV-infected plants or insects over the past two decades. The result was consistent with SRBSDV P7-2, which was not yet identified in SRBSDV-infected hosts [19]. The undetectable expression level of P7-2 might result from a low translational efficiency of the dicistronic mRNA [5], an exclusively early expression of the protein or a restricted expression to certain tissues. Recently, the relative mRNA expression level of the whole genome of SRBSDV in different hosts (including *Oryza sativa*, *Zea mays* and its insect vector *Sogatella furcifera*) was detected using RT-qPCR. The investigation confirmed that the mRNA level of P7-2 in each host is extremely low [20]. Although some assumptions about RBSDV P7-2 and its counterparts were obtained through the analysis of genome organization and sequence homology, no further specific information was found about them, and most of them belong to the functionally uncharacterized family DUF1139 (domain of unknown function).

In this study, a yeast two-hybrid (YTH) screening was conducted to investigate the interactions between RBSDV P7-2 and *Z*. *mays*. P7-2 was found to have an ability to interact with *Z*. *mays* SKP1 protein, an important component of SCF (SKP1/Cullin1/F-box protein/Rbx1) E3 ubiquitin ligase. The interaction was then confirmed *in planta* by bimolecular fluorescence complementation techniques (BiFC). Further experiments were developed to determine the respective region crucial for P7-2-SKP1 interaction. Agrobacterium-mediated transient suppression assay indicated that P7-2 has no obvious activity to suppress local RNA silencing. The results could be helpful to unravel the biological function of P7-2, and shed new lights on our understanding of the interaction between RBSDV and its host plants.

## Results

### P7-2 interacts with SKP1 from *Z*. *mays* in yeast

The potential functions of P7-2 in virus multiplication and viral pathogenicity on plants might be due to some unrevealed interactions between P7-2 and plant hosts. To investigate the function of P7-2, a two-hybrid screening was conducted. A plasmid expressing bait protein BD-P7-2 (P7-2 fused to the GAL4 DNA binding domain) was transformed into Y187. Making sure that the fused protein can be expressed in yeast and has no transcriptional activation or toxicity, BD-P7-2 was used as bait in two-hybrid screens of a cDNA library expressing *Z*. *mays* proteins fused to the GAL4 activation domain (AD).

**Figure 1:**
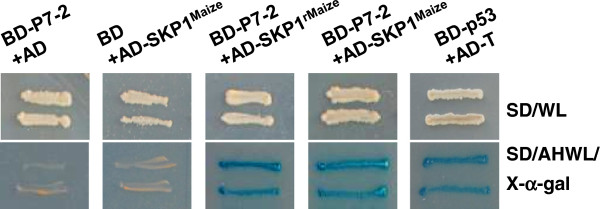
**Strong interaction between P7-2 and SKP1**^**Maize **^**in YTH system.** Yeast colonies containing the combinations of pGBKT7-S7-2/pGADT7-SKP1^Maize^ and pGBKT7-S7-2/pGADT7-SKP1^rMaize^ grew well on the selective medium and turned blue, as did yeast colonies containing the positive control pGBKT7-p53/pGADT7-T. pGADT7-SKP1^rMaize^ is the prey clone from the positive transformants obtained in the two-hybrid screens, harboring the mRNA sequence of SKP1, while pGADT7-SKP1^Maize^ is the construct that expresses the exact SKP1^Maize^ protein. Yeast transformed with pGBKT7-S7-2/pGADT7 or pGBKT7/pGADT7-SKP1^Maize^ used as negative controls were unable to grow.

Three independent transformants grew well under strong selective conditions and turned blue in the selective medium containing *α*-galactosidase. The interaction was also confirmed by *β*-galactosidase colony-lift filter assay. Sequence analysis indicated that the three transformants contained the same prey sequence, corresponding to the mRNA sequence of *Z*. *mays* SKP1-like protein 1A [GenBank: EU958896.1] [21], an ortholog of Arabidopsis Skp1-like protein (ASK1 proteins) and *Saccharomyces cerevisiae* SKP1 protein. The intact SKP1 ORF harbored in the mRNA sequence is 531 bp in length and encodes a protein (SKP1^Maize^) consisting of 176 amino acids. pGADT7-SKP1^Maize^, a plasmid expressing the exact SKP1^Maize^ protein fused to AD was then constructed and transformed into AH109. The YTH assay showed that P7-2 can interact with SKP1^Maize^ in yeast. No growth was observed for the negative controls (Figure 1).

SKP1 (S-phase kinase associated protein 1) is a core subunit of the SCF (SKP1/Cullin/F-box protein/Rbx1) family of E3 ubiquitin ligases, which play an essential role in the cellular ubiquitin-proteasome degradation system. The degradation process occurs as follows: ubiquitin molecules were firstly activated by the ubiquitin-activating enzyme (E1), and then transferred to E3 by the ubiquitin-conjugating enzyme (E2). The SCF E3 complexes covalently transfer the activated ubiquitin from E2 onto the substrates, and ultimately lead to the degradation of the poly-ubiquitinated substrates by the 26S proteasome. Through the degradation of particular targets, SCF complexes regulate many eukaryotic fundamental processes, such as cell cycle progression, transcriptional regulation, signal transduction, cellular trafficking and cell survival control [22]. And in the SCF complexes, SKP1 acts as a specific adapter linking the Cullin protein to diverse F-box proteins, which are able to recognize and bind substrate proteins through variable protein-protein interaction domains.

### Bimolecular fluorescence complementation assay confirmed the P7-2-SKP1^Maize^ interaction *in planta*

In order to exclude the possibility that a yeast protein could participate in the two-hybrid interactions and verify P7-2-SKP1^Maize^ interaction *in planta*, BiFC assay was performed. Two pairs of combinations that express P7-2 fused to YN (P7-2-YN) and SKP1^Maize^ fused to YC (SKP1^Maize^-YC), or P7-2 fused to YC (P7-2-YC) and SKP1^Maize^ fused to YN (SKP1^Maize^-YN) were constructed and delivered into *Nicotiana benthamiana* leaves via agro-infiltration.

**Figure 2:**
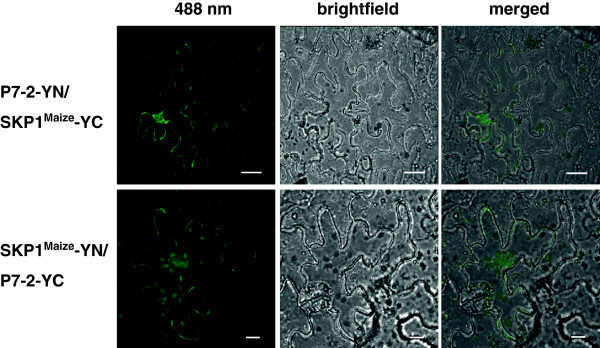
**BiFC visualization of P7-2-SKP1**^**Maize **^**interaction in agrobacterium-infiltrated *N*. *benthamiana *leaves.** Co-expression of P7-2-YN/SKP1^Maize^-YC or P7-2-YC/SKP1^Maize^-YN induced a weak fluorescence pattern. The signals were localized in or at the peripheries of the nuclei and the plasma membrane, and slight diffuse fluorescence was also observed in the cytoplasm. No YFP signals were detected for the negative controls. YFP was excited at 488 nm and emission was measured at 550–590 nm. The fluorescent, bright field and merged images are depicted in the upper, middle and bottom panels, respectively. Bars, 20 μm.

As expected, recovered YFP signals were visualized in both combinations in a weak pattern, localizing in or at the peripheries of the nuclei and the plasma membrane, and slight diffuse fluorescence was also observed in the cytoplasm (Figure 2). No YFP signals were detected for the negative controls following the co-expression of P7-2-YN/YC, P7-2-YC/YN, SKP1^Maize^-NE/YC or SKP1^Maize^-CE/YN (Additional file 1: Figure S1). As a key technique to visualize protein-protein interactions, BiFC assay provided the direct evidence for the P7-2-SKP1^Maize^ interaction *in planta*, which is consistent with the YTH result.

### P7-2 interacts with SKP1 proteins from other plants in yeast

There are many SKP1 homologs expressed in the animal and plant species, except protists, fungi, yeast, *Homo sapiens* and some vertebrates which have a single *SKP1*-*like* gene [22,23]. To detect whether the interaction is universal, different *SKP1*-*like* genes from dicotyledon and monocotyledon, including *N*. *benthamina*, *Arabidopsis thaliana*, *O*. *sativa* and *Saccharum sinense*, were isolated using RT-PCR method. And their interaction with P7-2 was tested via YTH assay. The obtained SKP1 proteins are ASK1 [GenBank: NM106245.4], ASK2 [GenBank: NM123584.4], NbSKP1 [GenBank: AF494084.1], OSK1 [LOC_Os11g26910], and SsSKP1 [GenBank: KF146307].

**Figure 3:**
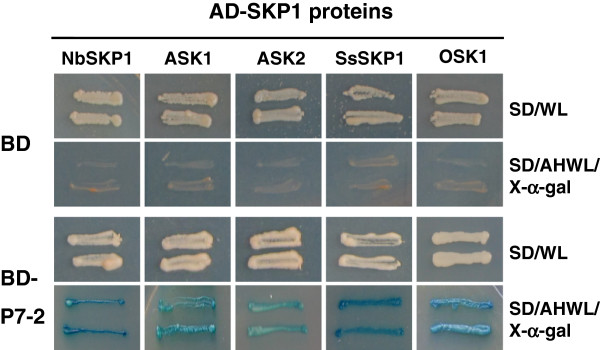
**Strong interaction between P7-2 and SKP1 proteins from other plants in YTH system.** Yeast colonies expressing BD-P7-2 with AD-ASK1, AD-ASK2, AD-NbSKP1, AD-SsSKP1, and AD-OSK1 were able to grow well on the selective medium and turned blue.

YTH analysis indicated that there is a strong interaction between P7-2 and each SKP1-like protein obtained above. Independent yeast colonies containing each combination grew well and turned blue in the selective medium containing *α*-galactosidase (Figure 3). The result suggested that the interaction between P7-2 and SKP1-like proteins is prevalent in the cellular environment. P7-2 might be a putative F-box protein (FBP) coded by RBSDV and involved in the ubiquitin proteasome (UP) system in the host.

### SKP1^Maize^ C-terminal region spanning residues 97 to 176 is necessary for P7-2-SKP1^Maize^ interaction

**Figure 4:**
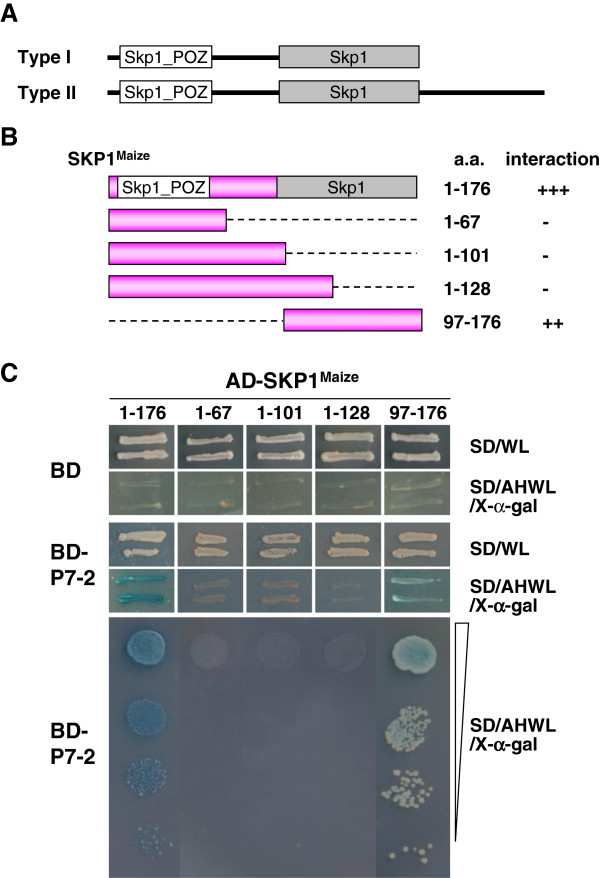
**Mapping of the crucial region of SKP1**^**Maize **^**involved in the P7-2-SKP1**^**Maize **^**interaction. (A)** Two types of SKP1 proteins. Both of the two types have two conserved domains (Skp1_POZ and Skp1) and two variable regions. Type II proteins have an additional C-terminal region. **(B)** Schematic representation of SKP1^Maize^ and its truncations in the study. The full-length SKP1^Maize^ (spanning residues 1 to 176) and its truncations are indicated by pink bars while the deleted regions by dashed lines. The numbers denote the amino acid positions of the proteins. The ability of different SKP1^Maize^ truncations to interact with P7-2 is indicated on the right (+, positive; -, negative). **(C)** The interaction between P7-2 and different SKP1 truncations in YTH assays. Yeast colonies expressing AD-SKP1^97-176^ with BD-P7-2 were able to grow on the selective medium and turned slight blue. No growth was observed in the yeast expressing BD-P7-2 with AD-SKP1^1-67^, AD-SKP1^1-101^ or AD-SKP1^1-128^.

The BLAST analysis indicated that all the SKP1 proteins obtained are SKP1 type I proteins, which have two conserved domains (i.e. Skp1_POZ and Skp1) and two variable regions (Figure 4A) [24]. The N-terminal Skp1_POZ domain and the C-terminal SKP1 domain are involved in the process of SKP1 tetramerization and dimerization, respectively.

To determine the region responsible for P7-2-SKP1^Maize^ interaction, four truncation derivatives were constructed, expressing AD-SKP1^1-67^, AD-SKP1^1-101^, AD-SKP1^1-128^, and AD-SKP1^97-176^, based on the domains of SKP1^Maize^. Their abilities to interact with P7-2 were investigated via YTH assay. Schematic representation of the different SKP1 truncation derivatives is shown in Figure 4B.

The YTH analysis indicated that AD-SKP1^1-67^, AD-SKP1^1-101^ and AD-SKP1^1-128^ completely lost the capabilities to interact with P7-2, and no growth was observed, as well as the negative control. AD-SKP1^97-176^, in which only 79 amino acids at the C-terminus were left, sustained the interaction with some decreased binding capability, compared to the whole SKP1 (Figure 4C). The results suggested that the C-terminal Skp1 domain is necessary to sustain the P7-2-SKP1 interaction, while N-terminal Skp1_POZ domain is helpful to stabilize the interaction in some degree. Our results are consistent with those of the crystal structure of the human F-box protein Skp2 bound to SKP1 [25]. The analysis of the crystal structure indicated that the last two helices of the BTB/POZ fold and the two helices of the C-terminal extension of Skp1 form the Skp2-binding site, corresponding to the SKP1^Maize^ fragment spanning residues 111 to 176 exactly.

### Both the middle and C-terminal region of P7-2 are greatly involved in P7-2-SKP1^Maize^ interaction

**Figure 5:**
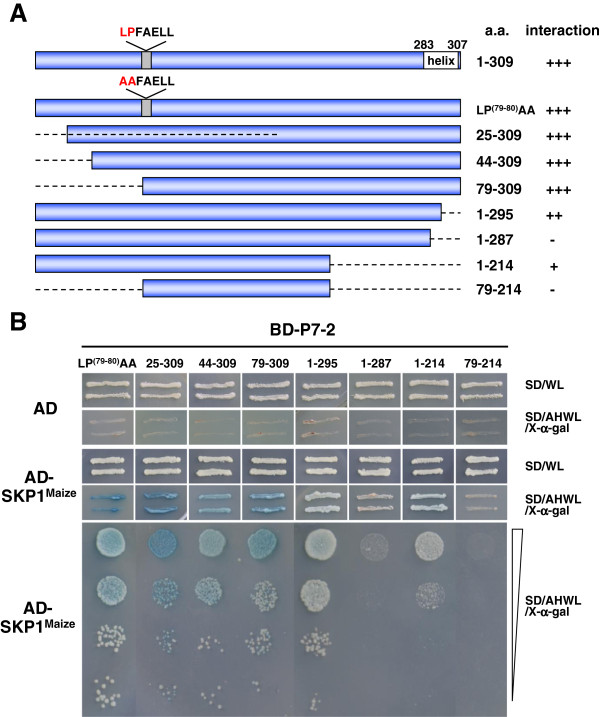
**Mapping of the crucial region of P7-2 involved in the interaction with SKP1**^**Maize**^**. (A)** Schematic representation of P7-2 and its truncations in the study. The full-length P7-2 (spanning residues 1 to 309) and its truncations are indicated by blue bars while the deleted regions by dashed lines. The predicted α-helix and the short sequence (LPFAELL) similar to F-box consensus sequence (LPxxI/L) were also indicated. The numbers denote the amino acid positions of the proteins. The ability of different P7-2 truncations to interact with SKP1^Maize^ is indicated on the right (+, positive; -, negative). **(B)** The interaction between different P7-2 truncations and SKP1^Maize^ in YTH assays. Yeast colonies expressing AD-SKP1^Maize^ with BD-P7-2^25-309^, BD-P7-2^44-309^, BD-P7-2^79-309^, and P7-2-LP^(79–80)^AA were able to grow well on the selective medium and turned blue. BD-P7-2^1-295^ and BD-P7-2^1-214^ showed decreased capability to interact with SKP1^Maize^. No growth was observed in the yeast expressing AD-SKP1^Maize^ with BD-P7-2^1-287^ and BD-P7-2^79-214^.

The strong interaction of P7-2-SKP1^Maize^ suggested that P7-2 might contain an F-box motif, a nonconserved N-terminally located ≈ 50 aa domain, through which FBPs binds SKP1. Thus, a protein secondary structure prediction of P7-2 was carried out, by the Jpred 3 and SCRATCH prediction server [26,27], as well as its counterparts in other fijiviruses. The prediction showed that there are no specific secondary structures at the N-termini of these proteins, while the region at the C-termini, spanning residues 283–307 as for P7-2, is predicted with high probability to be in an α-helical conformation. A collection of P7-2 truncation derivatives that express BD-P7-2^25-309^, BD-P7-2^44-309^, BD-P7-2^79-309^, BD-P7-2^1-295^, BD-P7-2^1-287^, BD-P7-2^1-214^ and BD-P7-2^79-214^ were constructed sequentially, and their capabilities to bind to SKP1^Maize^ were tested. Schematic representation of P7-2 and other mutant derivatives is shown in Figure 5.

The YTH analysis indicated that P7-2^25-309^, P7-2^44-309^ and P7-2^79-309^ are able to bind SKP1^Maize^ strongly, and the colonies expressing these combinations showed no obvious growth inhibition, compared to the intact P7-2. However, it is not the case when the truncations happened at the C-terminus. As is shown in Figure 5, P7-2^1-295^, lost 14 residues at C-terminus, has a slightly decreased activity to interact with SKP1. When 21 residues were deleted, P7-2^1-287^ showed a complete inability to attach SKP1. But when the deletion increased to 95 residues, P7-2^1-214^, which lost the whole α-helix comprised in C-terminus, recovered the interaction with a more weakened capability (Figure 5B).

A short sequence LPFAELL (residues 79–85 in P7-2) was found to show some similarity to the F-box consensus sequence (LPxxI/L), in which LP are the most highly conserved residues [28,29]. To investigate the effect of L^79^P^80^, a point mutant P7-2-LP^(79–80)^AA substituting two alanines for L^79^P^80^ was constructed. And P7-2-LP^(79–80)^AA was able to interact with SKP1^Maize^ strongly (Figure 5B).

The results suggested that the middle fragment of P7-2, spanning residues from 79–214 is crucial to sustain the P7-2-SKP1^Maize^ interaction. The putative α-helix region at the C-terminus, spanning residues 283–307, plays an important role to facilitate the interaction, and partial deletion of α-helix intends to induce some conformational changes so as to interrupt the association. L^79^P^80^ was not crucial during the interaction.

### Agrobacterium-mediated transient suppression assay indicated P7-2 has no obvious activity to suppress local RNA silencing

P7-2-SKP1 interaction suggested that P7-2 might be involved in the UP pathway. Up to now, many viruses have evolved different strategies to hijack the ubiquitination/proteolysis machinery to enhance their replication or counteract the host defense, by targeting cellular factors for degradation [30,31]. Considering the assumption that P7-2 might participate in virus multiplication and pathogenicity, its activity in suppressing post-transcriptional gene silencing (PTGS) was examined through Agrobacterium-mediated transient suppression assay.

As described previously [32], an Agrobacterium strain harboring a GFP expressing plasmid, pGDSmGFP, was mixed with another strain carrying the test plasmid, pGDS7-2His, and the mixture was co-infiltrated into leaves of *N*. *benthamiana*. The latter plasmid was able to express a His-tagged P7-2 protein. A construct expressing p19, the viral suppressor of RNA silencing (VSR) from *Tomato bushy stunt virus* (TBSV) was used as the positive control, while the empty vector as the negative control. The accumulation of GFP and P7-2 was detected by GFP antiserum and the specific anti-His-tag monoclonal antibodies, respectively.

**Figure 6:**
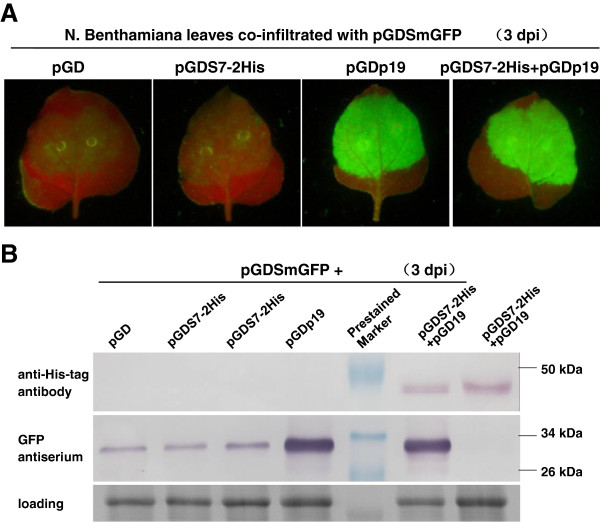
**PTGS suppression of GFP by RBSDV P7-2 in the *N*. *benthamiana *transient co-expression system. (A)** Agro-infiltration of *N*. *benthamiana* with pGDSmGFP and pGD (left), pGDS7-2His (middle), and pGDp19 (right). Photographs were taken under long-wavelength UV light at 5 dpi. **(B)** Western blot analysis using GFP polyclonal antiserum. The intensity of the GFP signals was consistent with that of GFP fluorescence. Total proteins stained with Coomassie blue were used as the loading control.

GFP fluorescence intensity in leaf regions co-infiltrated with pGDSmGFP and pGDS7-2His peaked at 3 days post-infitration (dpi), and declined rapidly over the next 2 days, as well as the negative combination of pGDSmGFP and pGD (Figure 6A). In contrast, when pGDSmGFP was co-infiltrated with pGDp19, the infiltrated patches displayed a bright green fluorescence even at 10 dpi. Western blot analysis confirmed the visual observation, for that the level of GFP protein accumulated were parallel to the intensity of GFP fluorescence (Figure 6B).

The expression level of P7-2 accumulated to a higher level when the protein was co-expressed with p19, while no accumulation was detected when p19 was absent. The result suggested that P7-2 is able to be expressed in the leaves of *N*. *benthamiana*, but the protein exhibits no activity to suppress local gene silencing and is degraded rapidly without the existence of p19. To confirm our assumption, bacteria harboring pGDS7-2His was co-infiltrated with those containing pGDp19 and pGDSmGFP. GFP fluorescence and protein accumulation were compared with the control treatment co-infiltrated with the combination of pGD19 and pGDSmGFP. As we expected, P7-2 was expressed and detected in the infiltrated patch, and the fluorescence intensity and GFP accumulation were equivalent to the combination control. Taken together, P7-2 has no obvious local VSR activity.

## Discussion

Since the intensified cultivation of the gramineous crops and the increase of the natural population of insect vectors these years, there has been an increase in plant-infecting fijiviruses producing severe damage on cultures. Investigating the functions of the viral proteins and elucidating the complicated process of the events in *Fijivirus* life cycle turn to be the focus of many researchers. Recently, some new functions of several proteins encoded by fijiviruses were reported, such as RBSDV P6, P5-1, P7-1 and their homologs [11,15,33]. However, the biological function of RBSDV P7-2 is still largely unknown, so as their counterparts in other fijiviruses. In this study, the interaction between P7-2 and the proteins of *Z*. *mays*, one of the main plant hosts of RBSDV, was investigated.

The YTH analysis and BiFC assay indicated that P7-2 is able to interact with SKP1 proteins. Additionally, we also found the association between P7-2 and AtElongin C (unpublished results), a SKP1-like protein that is also involved in the ubiquitin-mediated proteolysis [34]. As a key subunit of SCF ligase, SKP1 tethers the rest of complex to an FBP, a protein which functions as the substrate-recognition unit and confers substrate specificity to the complex [30,35]. The strong association suggests that P7-2 might be a potential FBP encoded by RBSDV and involved in the UP system.

UP system plays a very complex interaction with different viral replication cycle. Many viruses exploit the UP system to alter the cellular environment or disrupt host antiviral defenses, using different mechanisms [36]. Some of them directly express a novel E3 complex, whereas in others, viral FBPs are encoded to alter the specificity of host E3 ligases and serve the viral multiplication [30,37]. For example, herperviruses encode a viral E3 ligase that targets mediators of interferon signaling for degradation [38-40]. The Vif protein of *Human immunodeficiency virus type 1* (*HIV*-*1*) can target the retroviral complementary DNA deaminase APOBEC3G for degradation, by hijacking an ubiquitin ligase complex [41]. In another aspect, an active UP system may also provide some benefits to favor the replication of viruses. Several rotaviruses were found to require a functional UP system to replicate efficiently. In the experiments, the yield of infectious virus, the assembly of new viroplasms and the replication of the viral genome were decreased when the proteasome inhibitor MG132 was added, as well as the incorporation of viral proteins into viroplasms [42,43].

There are increasing reports that perturbation of host SCF E3 ligase is a common manner used by plant viruses. Viruses hijack the host SCF ligase by viral FBPs, which can bind to SKP1 via their F-box motifs and recruit specific target proteins through other domains, and finally lead to the degradation of the target proteins [36]. The first example of this strategy is CLINK, an FBP encoded by *Faba bean necrotic yellow virus* (FBNYV) [44]. CLINK interacts with SKP1 through its F-box motif and associate with the retinoblastoma tumor-suppress protein pRB via an LxCxE motif. Through modification of pRB activity, the virus affects plant cell cycle regulation to promote its replication [45]. Another example is P0 protein, the VSR of polerovirus. P0 binds SKP1 by means of its conserved F-box motif LPxxI/L. Point mutations in the F-box motif abolished the P0-SKP1 interaction, diminished virus pathogenicity, and inhibited its VSR activity [46]. Further investigation unveiled that P0 targets AGO1 protein, the key component of RNA-induced silencing complex (RISC), and finally triggers its decay *in planta*[47,48]. In our study, P7-2 did not possess obvious local VSR activity as P0. Thus, the P7-2-SKP1 interaction raises some interesting questions. What is the biological role of the interaction? Is P7-2 a real F-box protein and contained in a SCF complex? What is its target protein? Furthermore, trancriptome and comparative gene expression analysis of *S*. *furcifera* in response to SRBSDV indicated that SRBSDV infection greatly perturbs its primary metabolism and the UP pathways. During the candidate transcripts elicited, SKP1 was upregulated in viruliferous planthoppers [49]. The findings hinted that UP pathway of the insect hosts is greatly involved in the process of virus infection. Our recent work found that P7-2 enhanced the pathogenicity of *Potato virus X* (PVX) in *N*. *benthamiana* via a heterologous expression strategy. Intriguingly, Western blot analysis indicated that, compared to the other treatments, the accumulation of the PVX coat protein in the systemic leaves of PVX-S7-2 treatment did not increased, but decreased to a very low level (Additional file 2: Figure S2, unpublished results). Further experiments are conducted to elucidate the biological significance of P7-2 and the interaction.

Generally, a typical FBP always contains an F-box motif at its N-terminus and additional protein-protein interaction motifs at its C-terminus, such as Trp-Asp (WD), leucine-rich repeats (LRR), zinc fingers or Kelch repeats, which are responsible for the interaction with SKP1 and the target substrates, respectively [30,37]. However, as a member of DUF1139 family, no typical motifs were found out in P7-2. The failure to identify the F-box motif might be due to its loosely conserved amino acid consensus, small insertions, and limitations of the recent search algorithms, most of which is established on the sequences of the F-box proteins predicted in yeast, nematode and Arabidopsis [28]. Notably, both P7-2 and its counterparts are leucine-rich protein. As the most abundant amino acid, leucine accounts for 12.3%, 12.6%, 12.3%, 10.3%, 11.7% and 11.2% in the composition of RBSDV P7-2, MRDV P6-2, SRBSDV P7-2, MRCV P7-2, FDV P7-2 and OSDV P7-2, respectively, far more than any other kind of amino acids. Although no conventional LRRs were found, the common feature hinted that there might be some leucine-related structures embedded in P7-2 and its homologs.

As is known to us, RBSDV P7-2 was expressed in the plant and insect hosts at a very low level, which is generally considered to be due to the low translational efficiency of the dicistronic mRNA [5]. It is consistent with the expression of SRBSDV P7-2 in virus hosts and MRCV P7-2 in Sf9 insect cells [20,50]. Many FBPs were found to be short-lived or expressed at a very low level, such as Cdc4p within the *Saccharomyces cerevisiae* SCF^Cdc4p^ and polerovirus P0 [51,52]. As FBPs are located in the enzymatic interface to link substrates for attaching ubiquitin, they become targets as well. Once an FBP is ubiquitinated and degraded, SCF complexes disassembly and the other components might be recycled to form new SCF complexes [30]. Based on our result, the proteolysis caused by its self-ubiquitination might be one possibility.

As a core component of SCF ligase, SKP1 family mediates targeted protein degradation for the regulation of diverse plant-specific process. Many plant species possess a large number of SKP1 homologs, divided into type I and type II. For example, there are 21 SKP1 genes in the Arabidopsis genome, among which 19 are type I and 2 are type II genes. The rice genome encodes 32 SKP1 genes at least, and 28 belong to type I genes [24]. Although possessing a significant degree of functional redundancy, different SKP1 family members also display multiple divergences in gene spatial and temporal expression, protein localization, interaction networks and some other aspects, and are engaged in the formation of specific SCF complexes to regulate different biological processes [23]. In our study, P7-2 was found to interact with six SKP1 proteins, all of which belong to type I genes. Whether P7-2 interacts with SKP1 proteins from type II genes is still not determined. Further investigation will be focused on the interaction between P7-2 and different SKP1 proteins. New information may provide better understanding of the biological functions of P7-2-SKP1 interaction.

## Conclusions

To date, the function of RBSDV P7-2 is largely unknown, as well as its counterparts in other fijiviruses, and all of them belong to DUF1139 family. Our results indicated that P7-2 is able to interact with different SKP1 proteins, the key component of SCF ubiquitin ligase. The respective regions of SKP1 and P7-2 necessary to sustain the interaction were also determined. This is the first report linking a *Fijivirus* protein to a component of the UP system. Our findings provide some new clues to understand the function of P7-2 and help us gain further insight into the *Fijivirus*-host interaction.

## Methods

### General

*N*. *benthamiana* plants were grown in a controlled environmental climate chamber at 23°C under 1,000 lumens with a 16-hour daylight regimen. *Agrobacterium tumefaciens* strain EHA105 was grown on LB agar containing 50 g/ml rifampin. The yeast strains, *S*. *cerevisiae* AH109 and Y187, and the yeast vectors, pGBKT7 and pGADT7, as well as the positive control plasmids, pGBKT7-p53 and pGADT7-T, were used for YTH analyses (Clontech). RBSDV S7 (GenBank: AF397894) full-length cDNA clone (pHbm-S7) was maintained in our lab [7]. BiFC vectors, pSPYNE-35S and pSPYCE-35S, were generously provided by Professor Jörg Kudla, Universität Müneter, Germany. The binary expression vectors pGD, pGDSmGFP and pGDp19 were obtained from Professor Andrew O. Jackson of the University of California at Berkeley. The *Z*. *mays* cDNA library was kindly provided by Professor Zaifeng Fan.

### Construction of recombinant plasmids

To generate yeast plasmid for the two-hybrid assay, P7-2 ORF was amplified from pHbm-S7, using primers PS7-6/PS7-9. PCR products were ligated to pMD19-T to get the intermediate pMD19-T-S7-2. The clone was digested with *Nco*I/*Bam*HI, and then ligated into the same sites of pGBKT7 to generate pGBKT7-S7-2. Using pMD19-T-S7-2 as the template, inverse PCR and site-mutagenesis strategy was adopted to obtain the plasmid harboring the P7-2-LP^(79–80)^AA segment. The clone was cut by *Nco*I/*Bam*HI and the liberated fragment was ligated into *Nco*I/*Bam*HI-digested pGBKT7 to generate pGBKT7-S7-2-LP^(79–80)^AA. Vectors expressed P7-2 truncations (P7-2^25-309^, P7-2^44-309^, P7-2^79-309^, P7-2^1-295^, P7-2^1-287^, P7-2^1-214^ and P7-2^79-214^) as bait were created by similar strategies using the appropriate specific primers (Table 1). To generate pGADT7-SKP1^Maize^, SKP1^Maize^ ORF was amplified from the screened prey plasmid harboring the whole SKP1 mRNA, using primers MSKP1-1/MSKP1-2. PCR products were ligated to pMD19-T to get pMD19-T-SKP1^Maize^. The clone was then cut by *Bam*HI/*Xho*I and the liberated fragment was ligated into *Bam*HI/*Xho*I-digested pET30a to obtain pET30a-SKP1^Maize^. The plasmid was digested by *Nco*I/*Xho*I and the liberated fragment was ligated into *Nco*I/*Xho*I-digested pGADT7 to yield pGADT7-SKP1^Maize^. The yeast plasmids expressing SKP1^Maize^ truncations (SKP1^1-67^, SKP1^1-128^ and SKP1^97-176^) were amplified from pMD19-T-SKP1^Maize^, using specific primers (Table 1). And the PCR products were digested with *Eco*RI/*Bam*HI, and then ligated into the same sites of pGADT7 to create the corresponding constructs. pGADT7-SKP1^Maize^ was treated by *Sal*I/*Xho*I, then the larger fragment produced was extracted and self-ligated to create pGADT7-SKP1^1-101^. To obtain the prey vectors expressing SKP1 proteins from *A*. *thaliana*, *N*. *benthamiana*, *O*. *sativa* and *S*. *sinense*, total RNA of the leaves of these plants was isolated using Trizol reagent (Invitrogen) according to the manufacturer’s protocol. And first-strand cDNA synthesis was carried out with M-MLV reverse transcriptase (Promega). Target SKP1 genes were amplified using the specific primers and ligated into pGADT7.

**Table 1:** Primers used for PCR amplification

**Primer**	**Sequence (5′ → 3′)**^**a**^	**Locations**^**b **^**and modifications**
PS7-6-F	CTAG ccatgg *GA* ATGAATTACACTTTAAGTG	1aa; *Nco*I
PS7-9-R	CG ggatcc *tcacta* TTAAGAATTCAGTATC	Full-length reverse primer with stop codon; *Bam*HI;
PS7-14-F	GAACTC ggatcc ATGAATTACACTTTAAG	1aa; *Bam*HI;
PS7-15-R	CCG ctcgag TTAAGAATTC AGTATC	Full-length reverse primer with stop codon; *Xho*I;
PS7-16-R	CCC aagctt *tta***GTGGTGGTGGTGGTGGTG** AGAATTCAGTATCTTTTTG	Full-length reverse primer without stop codon; *Hin*dIII; six His were introduced;
PS7-17-R	CCG ctcgag AGAATTCAGTATCTTTTTG	Full-length reverse primer without stop codon; *Xho*I;
PS7-18-R	T ***GC*** AGGAACG TCGAATGAATC	79aa; GC substitute for AA;
PS7-19-F	***G*** CTTTTGCTGAGTTGCTTGATC	80aa; G substitute for C;
TS7-23-F	ggatcc gtcgac ccatgg *GA* CCCGAAATTAATCTTGTC	25aa; *Bam*HI, *Nco*I, *Sal*I;
TS7-25-F	ggatcc gtcgac ccatgg *GA* GTGAATTTACTATCTGAT	44aa; *Bam*HI, *Nco*I, *Sal*I;
TS7-27-F	ggatcc gtcgac ccatgg *GA* TTACCTTTTGCTGAGTTG	79aa; *Bam*HI, *Nco*I, *Sal*I;
TS7-8-R	CG ggatcc ggtacc *tcacta* TTAAGAATTCAGTATCT	Full-length reverse primer with stop codon; *Bam*HI, *Kpn*I;
TS7-21-F	ggatcc gtcgac ccatgg *GA* ATGAATTACACTTTAAGT	1aa, *Bam*HI, *Sal*I, *Nco*I;
TS7-12-R	CG ggatcc ggtacc *tta* ACTCTTAAATATCAAAG	295aa; *Bam*HI, *Kpn*I;
TS7-14-R	CG ggatcc ggtacc *tta* AAACAGAGAATACCAATAATC	287aa; *Bam*HI, *Kpn*I;
TS7-16-R	CG ggatcc ggtacc *tta* GGGTATCATACTTAATTTTCC	214aa; *Bam*HI, *Kpn*I;
MSKP1-1-F	CG ggatcc ATGGCCGCCGAGGGC	1aa; *Bam*HI;
MSKP1-2-R	CCG ctcgag CTCGAAGGCCCACTG	Full-length reverse primer without stop codon; *Xho*I;
MSKP1-3-F	CG gaattc ATGGCCGCCGAGGGC	1aa; *Eco*RI;
MSKP1-4-R	CG ggatcc TACGTGCTTGTTGCAG	67aa; *Bam*HI;
MSKP1-5-F	CG gaatcc GAGGACCTCAAGAAC	97aa; *Eco*RI;
MSKP1-6-R	CG ggatcc ACCCTTGATGTTCAGATAG	128aa; *Bam*HI;
MSKP1-8-F	CCG ctcgag *CT* ATGGCCGCCGAGGG	1aa; *Xho*I;
MSKP1-9-R	CG ggatcc CTCGAAGGCCCACTG	Full-length reverse primer without stop codon; *Bam*HI;
MSKP1-10-F	CCC aagctt *CG* ATGGCGGCCGAGGGCGAG	1aa; *Hin*dIII;

^a^Introduced restriction endonuclease sites are in lower case. Two extra nucleotides (italicized) were added to allow in-frame expression of fusion proteins of interest. The italicized nucleotides in lower case denote the stop codon added, while three mutant sites are denoted by boldfaced capitals in italic.

^b^Numbered according to the amino acid sequence of P7-2 or SKP1^Maize^. The F or R designation in the primer names denotes that the primer is a forward (5′) or reverse (3′) primer, respectively.

To obtain binary vectors for BiFC, P7-2 ORF was amplified from pHbm-S7, using primers PS7-12/PS7-13. PCR products were reversely ligated into pMD19-T. The clone was digested with *Xba*I/*Bam*HI, and then ligated into the same sites of pSPYNE-35S and pSPYCE-35S to generate vectors expressing P7-2-NE and P7-2-CE. pMD19-T-SKP1^Maize^ (obtained above) was digested with *Bam*HI/*Xho*I, and the liberated fragment was ligated into BiFC vectors to generate plasmids expressing SKP1^Maize^-NE and SKP1^Maize^-CE.

To obtain binary vectors for Agrobacterium-mediated transient suppression assay, P7-2 ORF was amplified from pHbm-S7, using primers PS7-6/PS7-16. PCR products were forwardly ligated into pMD19-T. The clone was digested with *Sal*I/*Bam*HI, and then ligated into the same sites of pGD to generate pGDS7-2His.

All the primers used were shown in Table 1. And all the regions generated by PCR were verified by sequencing, and the recombinant plasmids were confirmed by restriction analyses.

### YTH and *β*-galactosidase assays

Yeast transformation, two-hybrid assay and β-galactosidase colony-lift filter assay were performed using the Matchmaker GAL4 Two-Hybrid System3 (Clontech), according to the manufacturer’s protocols. Cotransformants were plated on synthetic defined (SD) minimal medium minus adenine, histidine, leucine, and tryptophan (SD/-Ade/-His/-Leu/-Trp), and positive yeast colonies that could grow on the auxotrophic medium were lysed in liquid nitrogen and then tested for *β*-galactosidase activity as mentioned in the *β*-galactosidase colony-lift filter assay.

### BiFC assay in *N*. *benthamiana* leaves using confocal laser scanning microscopy

BiFC assay was conducted according to Wang et al. [14]. Different binary plasmids were transformed into *Agrobacterium tumefaciens* EHA105 by a freeze-thaw method. Cultures of EHA105 harbouring a relevant binary plasmid were grown in LB medium containing rifampicin (50 g/ml) and kanamycin (100 g/ml) at 28°C for 16 h. *Agrobacterium* cultures containing the BiFC constructs and the pGDp19 plasmid were resuspended at a final OD_600_ of 0.5:0.5:0.3, with infiltration medium (10 mM MES, pH 5.6, 10 mM MgCl_2_, 150 mM acetosyringone). The cells were incubated at room temperature for 2 to 4 h, and then infiltrated into 5-6-week-old *N*. *benthamiana* leaves. Underside epidermal cells of tobacco infiltrated leaves were assayed for fluorescence at 48–96 h after infiltration. Fluorescence analysis was performed using a Nikon ECLIPSE TE2000-E inverted fluorescence microscope equipped with a Nikon D-ECLIPSE C1 spectral confocal laser scanning system. YFP signal was detected with an excitation at 488 nm and emission capture at 550–590 nm.

### Agrobacterium-mediated transient suppression assay and GFP imaging

Agro-infiltration and GFP imaging were performed as described by Han et al. [32]. *Agrobacterium* culture containing the test plasmids and pGDSmGFP were resuspended at a final OD_600_ of 0.5:0.5. Plants were illuminated with a 100 W hand-held long-wave ultraviolet lamp for photography and images were taken with a Nikon 4500 digital camera.

### SDS-PAGE and Western blot analysis

SDS-PAGE and Western blot analysis were performed as described [32,52]. The inoculated region of tobacco leaves was collected and ground to powder in liquid nitrogen. The samples were mixed with 2 × gel loading buffer, boiled for 5 min and then centrifuged for 5 min at 6,000 × g. Then the samples were separated by 12.5% SDS-PAGE, and transferred to Hybond-C membranes. The membranes were blocked overnight in 5% nonfat dried milk in TBST buffer (150 mM NaCl, 10 mM Tris–HCl, pH 8.0, 0.05% Tween-20) and then incubated for 4 h at room temperature with specific polyclonal antibodies raised against GFP (1:3,000 dilution). The membranes were washed with TBST buffer for three times and incubated with 1:5000 diluted Protein A-alkaline phosphatise (Sigma) in TBST. The GFP signal was detected with BCIP/NBT substrate.

## Competing interests

The authors declare that they have no competing interests.

## Authors’ contributions

QW carried out most of the experiments and wrote the manuscript. TT and XC anticipated the construction of the recombinants. YH anticipated the YTH assay. ZF provided the *Z*. *mays* cDNA library. CH, DL and JY conceived of the study and participated in its design and coordination. All authors read and approved the final manuscript.

## Supplementary Material

Additional file 1: Figure S1 The negative controls for the BiFC assay. No YFP signals were detected for the negative controls following the co-expression of P7-2-YN/YC, P7-2-YC/YN, SKP1^Maize^-NE/YC or SKP1^Maize^-CE/YN. Bars, 20 μm. Click here for file

Additional file 2: Figure S2 Phenotypic observations and molecular analysis of plants inoculated with chimaeric PVX vectors harbouring different RBSDV genes. (A) Systemic symptoms and Western blot analysis of PVX CP in the infiltrated and systemic leaves of *N*. *benthamiana* at 7 dpi. The treatments of PVX-S7-2, PVX-S9-1 and PVX-S9-2 triggered more severe PVX symptoms in the systemic leaves of *N*. *benthamiana*, while the other treatments showed similar symptoms as that of the empty vector. However, the accumulation level of the viral coat protein (PVX CP) in the systemic leaves of PVX-S7-2 treatment did not increased, but decreased in some degree. The CP accumulation levels of all the other treatments are equivalent to the control. (B) Systemic symptoms and Western blot analysis of PVX CP in the systemic leaves of *N*. *benthamiana* at 15 dpi. The viral symptoms of PVX-S7-2 treatment were more severe than the other treatments; however, the accumulation of PVX CP was decreased to a much lower level. Click here for file
