# Supplementary figures and images for: Nonstructural protein P7-2 encoded by Rice black-streaked dwarf virus interacts with SKP1, a core subunit of SCF ubiquitin ligase
Source: Virol J. 2013 Nov 1;10:325. doi: 10.1186/1743-422X-10-325 (PMC3819663; doi:10.1186/1743-422X-10-325)

## Slide 1
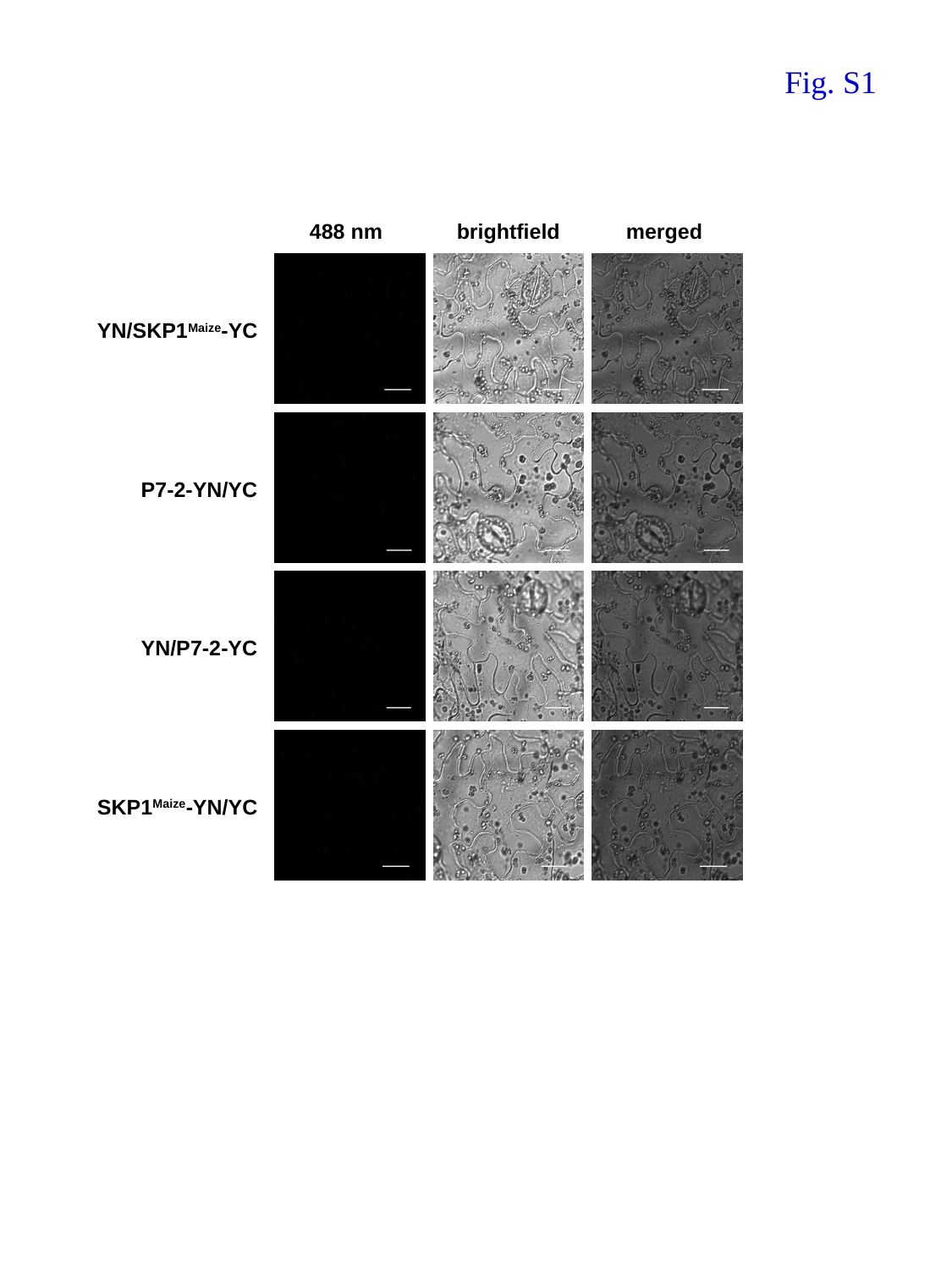

Fig. S1
488 nm
brightfield
merged
YN/SKP1Maize-YC
P7-2-YN/YC
YN/P7-2-YC
SKP1Maize-YN/YC

Supplement: Additional file 1: Figure S1 — The negative controls for the BiFC assay. No YFP signals were detected for the negative controls following the co-expression of P7-2-YN/YC, P7-2-YC/YN, SKP1Maize-NE/YC or SKP1Maize-CE/YN. Bars, 20 μm. [file 1743-422X-10-325-S1.ppt]
