# Supplementary material for: Nonstructural protein P7-2 encoded by Rice black-streaked dwarf virus interacts with SKP1, a core subunit of SCF ubiquitin ligase
Source: Virol J. 2013 Nov 1;10:325. doi: 10.1186/1743-422X-10-325 (PMC3819663; doi:10.1186/1743-422X-10-325)
Supplement: Additional file 2: Figure S2 — Phenotypic observations and molecular analysis of plants inoculated with chimaeric PVX vectors harbouring different RBSDV genes. (A) Systemic symptoms and Western blot analysis of PVX CP in the infiltrated and systemic leaves of N. benthamiana at 7 dpi. The treatments of PVX-S7-2, PVX-S9-1 and PVX-S9-2 triggered more severe PVX symptoms in the systemic leaves of N. benthamiana, while the other treatments showed similar symptoms as that of the empty vector. However, the accumulation level of the viral coat protein (PVX CP) in the systemic leaves of PVX-S7-2 treatment did not increased, but decreased in some degree. The CP accumulation levels of all the other treatments are equivalent to the control. (B) Systemic symptoms and Western blot analysis of PVX CP in the systemic leaves of N. benthamiana at 15 dpi. The viral symptoms of PVX-S7-2 treatment were more severe than the other treatments; however, the accumulation of PVX CP was decreased to a much lower level. [file 1743-422X-10-325-S2.ppt]

## Slide 1
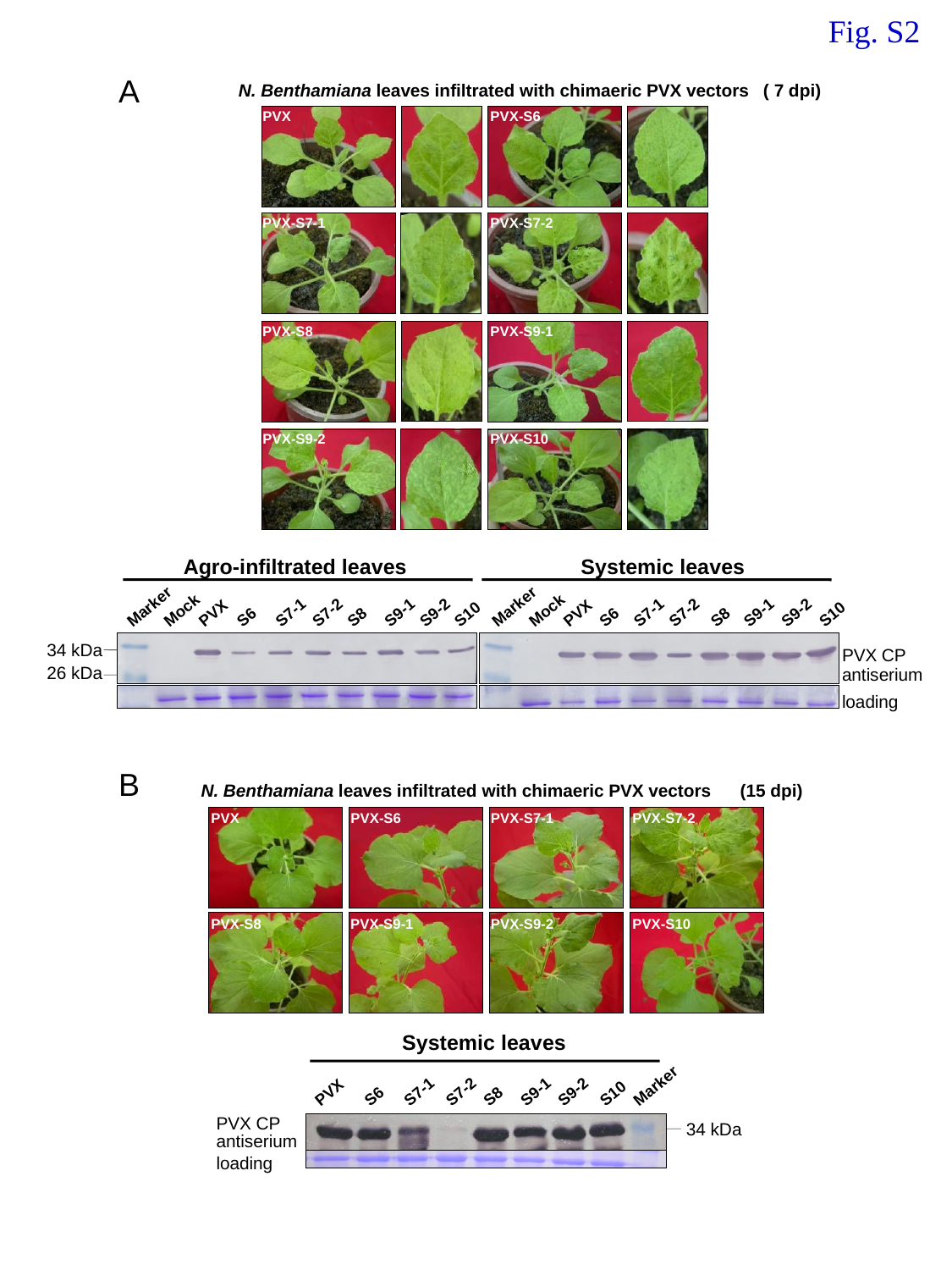

Fig. S2
A
N. Benthamiana leaves infiltrated with chimaeric PVX vectors ( 7 dpi)
PVX
PVX-S6
PVX-S7-1
PVX-S7-2
PVX-S8
PVX-S9-1
PVX-S9-2
PVX-S10
Agro-infiltrated leaves
Systemic leaves
S7-1
PVX
Marker
Marker
S7-2
Mock
Mock
S9-1
PVX
S6
S9-2
S9-2
S6
S7-2
S10
S10
S7-1
S8
S8
S9-1
34 kDa
26 kDa
PVX CP
antiserium
loading
B
N. Benthamiana leaves infiltrated with chimaeric PVX vectors (15 dpi)
PVX
PVX-S6
PVX-S7-1
PVX-S7-2
PVX-S8
PVX-S9-1
PVX-S9-2
PVX-S10
Systemic leaves
PVX
Marker
S7-2
S9-1
S7-1
S6
S9-2
S10
S8
34 kDa
PVX CP
antiserium
loading
